# Presence records of *Aedes vexans* (Diptera, Culicidae) in Germany

**DOI:** 10.3897/BDJ.14.e191869

**Published:** 2026-06-25

**Authors:** Peter Pothmann, Doreen Werner, Helge Kampen, Hans-Hermann Thulke

**Affiliations:** 1 Leibniz-Zentrum für Agrarlandschaftsforschung, Müncheberg, Germany Leibniz-Zentrum für Agrarlandschaftsforschung Müncheberg Germany; 2 Helmholtz Centre for Environmental Research - UFZ, Leipzig, Germany Helmholtz Centre for Environmental Research - UFZ Leipzig Germany; 3 Technische Universität Dresden, Dresden, Germany Technische Universität Dresden Dresden Germany; 4 Friedrich-Loeffler-Institut, Greifswald - Insel Riems, Germany Friedrich-Loeffler-Institut Greifswald - Insel Riems Germany

**Keywords:** observation, occurrence, distribution

## Abstract

**Background:**

*Aedes vexans*, a widespread mosquito species in Germany, is a major nuisance pest and a demonstrated or putative vector of numerous pathogens affecting humans and animals. To document its spatial and temporal occurrence, both passive and active mosquito monitoring programmes have been conducted across Germany.

Passive monitoring is carried out through the citizen-science project “Mückenatlas”, in which volunteers collect and submit mosquito specimens from across the country. All citizen-collected specimens are subsequently identified by professional entomologists. Active monitoring is performed by experts using a variety of standardised entomological methods, including trapping, aspirating and netting adults, as well as sampling larvae and pupae by dipping and sieving. Portions of the resulting dataset have already been used in scientific publications.

**New information:**

This data paper presents a comprehensive dataset on the occurrence of *Aedes vexans* in Germany, comprising 6,422 records collected between 26.04.2011 and 21.11.2023. Of the total occurrence records, 2963 originate from citizen-science contributions, while the remainder derive from active monitoring efforts. The dataset documents 225,565 individual mosquitoes from 2,784 distinct locations.

## Introduction

Mosquitoes are of major public and animal health relevance, as they may act as nuisance pests and vectors of a range of pathogens (Becker et al. 2020). Changes in climate, land use and global connectivity have facilitated the spread and establishment of both native and invasive mosquito species across Europe, including Germany (Walther and Kampen 2017, Franklinos et al. 2019). Spatially extensive long-term monitoring is therefore essential to understand mosquito distribution patterns, assess establishment risks and support vector surveillance and management strategies.

The dataset used in this study represents a combination of active and passive mosquito monitoring conducted across Germany. The passive component is based on the independent citizen-science initiative “Mückenatlas”, in which members of the public collect and submit mosquito specimens. This is complemented by active monitoring, conducted by professional entomologists.

The dataset presented here includes 6422 presence-only occurrence records of Aedes (Aedes) vexans (Meigen, 1830), a widespread mosquito species in Germany (Mohrig 1969). Although native to Europe, it is of particular interest due to mass occurrence events, its floodplain-associated ecology and its potential role as a vector of pathogens (Kampen and Walther 2018, Martinet et al. 2019, Becker et al. 2020). Understanding the spatial distribution is also important for modelling habitat suitability of *Ae. vexans* (Pothmann et al. 2025). However, as of 11 February 2026, only 53 occurrence records from Germany were registered in the Global Biodiversity Information Facility (GBIF), highlighting the limited availability of standardised data for this species.

The presented occurrence data have already been used in peer-reviewed studies addressing mosquito distribution modelling, habitat suitability and vector ecology. Previous work used *Aedes vexans* occurrence data within a machine-learning framework to help characterise the climatic niche of the invasive species Aedes (Finlaya) japonicus (Theobald, 1901) under conditions of limited occurrence data for the target species (Kerkow et al. 2020), to develop combined climate-habitat mosquito models (Wieland et al. 2021) and to evaluate automated feature selection for mosquito distribution modelling (Wieland et al. 2017). In addition, subsets of the data have contributed to studies on West Nile virus vectors in Germany (Kampen et al. 2020) and to a recent inventory of the German mosquito fauna (Werner et al. 2020).

Beyond species-specific applications, the “Mückenatlas” data have been analysed to assess structural and behavioural biases inherent to opportunistic citizen-science datasets. The dataset presented here represents a subset of the broader “Mückenatlas” data. Pernat et al. (2021) investigated temporal, spatial and environmental drivers of submission patterns in the “Mückenatlas”, demonstrating that submission numbers are strongly influenced by human population density, regional factors and environmental conditions, while also reflecting participant behaviour rather than true mosquito abundance alone. A follow-up study by Pernat et al. (2022) further quantified the influence of mass media coverage on citizen participation, showing that both the intensity and framing of media reports substantially affect submission numbers across time and space. Although these studies considered multiple mosquito species and the “Mückenatlas” dataset as a whole, the identified biases are equally relevant for *Ae. vexans* occurrence records and should be considered when interpreting and modelling the data presented here.

## Project description

### Title

Presence Records of *Aedes vexans* (Diptera, Culicidae) in Germany

### Personnel

Doreen Werner, Helge Kampen

### Funding

The work was financially supported by the German Ministry of Agriculture, Food and Regional Identity (BMLEH) through the Federal Office for Agriculture and Food (BLE), grant numbers 2810HS022, 2819104115, 2819104615 and 2818SE001 and by the Robert-Koch-Institute, Germany, grant number 1362/1-982.

## Sampling methods

### Study extent

Data collection was carried out within the administrative boundaries of Germany. The sampling frequency and exact locations were irregular and potentially opportunistic, depending on the monitoring approaches. Active monitoring was constrained by access, personnel and financial resources, while passive monitoring relied on random observations, resulting in uneven spatial and temporal coverage.

### Sampling description

Mosquitoes were collected using both active and passive surveillance approaches. Passive sampling was conducted through the indipendent citizen-science project “Mückenatlas”, launched in 2012, in which citizens submitted mosquitoes from their private surroundings for identification (Werner et al. 2020). All submitted specimens were subsequently identified by skilled entomologists.

Active sampling of adult mosquitoes was conducted during the vegetative seasons (April–October) using CO₂-baited BG Sentinel (Biogents, Regensburg, Germany) and EVS (encephalitis virus surveillance) traps (BioQuip Products, Compton, CA, USA) at numerous sites across Germany. These traps were typically activated for 24 hours per week and could yield multiple individuals. Additional adults were collected by aspirating resting females in animal shelters and zoos, sampling hibernation sites and netting from vegetation or animal and human bait. Larvae and pupae were sampled from natural and artificial breeding sites using the dipping technique(Werner et al. 2020).

Morphologically, all mosquitoes were identified as adults. Thus, collected aquatic stages were often carried along in water from their breeding sites and kept until adult emergence. Morphological identification of adults followed standard keys (Mohrig 1969, Schaffner et al. 2001, Becker et al. 2010). Damaged adults and individuals belonging to cryptic or closely-related species or those that could not be identified, based on morphological characters, were identified using CO1 (cytochrome *c* oxidase subunit 1) PCR, sequencing (Folmer et al. 1994, Hebert et al. 2003), as well as species-specific PCR assays (Proft et al. 1999, Rudolf et al. 2013, Kronefeld et al. 2014). After identification, a database record was created in the German mosquito database CulBase for each occurrence, including information on the federal state, geographic coordinates (latitude and longitude), sampling time or period, species identification, number of individuals collected, sex, developmental stage, habitat type and collection method. For a detailed description of the sampling design, data collection and results, see Werner et al. (2020).

### Quality control

The reported years were verified to exclude potential typographical errors and only data from years in which monitoring was actually conducted were retained. The coordinates were checked against federal state boundaries using GADM version 4.1. Two points appeared to lie slightly outside the reported federal states according to GADM. However, manual inspection revealed that the GADM dataset inaccurately represents the exact state boundaries in these cases. Therefore, the original data entries are considered correct.

### Step description

In the Sampling Description section, the methods used to collect mosquito occurrences are described in detail. All collected occurrence records are stored in the CulBase database. From this database, all records of *Ae. vexans* were extracted for publication in this dataset.

## Geographic coverage

### Description

N/A

### Coordinates

47.535587 and 54.776126 Latitude; 6.04961 and 14.996527 Longitude.

## Taxonomic coverage

### Taxa included

**Table:** 

Rank	Scientific Name	
kingdom	Animalia	
phylum	Arthropoda	
subphylum	Hexapoda	
class	Insecta	
order	Diptera	
family	Culicidae	
subfamily	Culicinae	
tribe	Aedini	
genus	Aedes	
subgenus	Aedes (Aedimorphus)	
species	Aedes vexans	

## Traits coverage

### Data coverage of traits

The dataset does not contain any information on species traits.

## Temporal coverage

### Notes

2011-04-26 through 2023-11-21

## Collection data

### Collection name

German mosquito database CulBase

## Usage licence

### Usage licence

Creative Commons Public Domain Waiver (CC-Zero)

### IP rights notes

This work is licensed under a Creative Commons Attribution Non-Commercial (CC-BY-NC 4.0) Licence.

## Data resources

### Data package title

Presence records of *Aedes vexans* (Diptera, Culicidae) in Germany

### Resource link

https://doi.org/10.15468/8xt7eh

### Number of data sets

1

### Data set 1.

#### Data set name

Presence records of *Aedes vexans* (Diptera, Culicidae) in Germany

#### Data format

Simple Darwin Core. Version: 13.09.2023

#### Download URL

https://ipt.pensoft.net/resource?r=aedesvexansgermany&v=1.4

#### Description

*Ae. vexans*, one of the most widespread and abundant mosquito species in Germany, is a demonstrated or putative vector of numerous pathogens of humans and animals. To document its spatial and temporal occurrence, we compiled a comprehensive dataset based on both passive and active mosquito monitoring. The dataset comprises 6,422 occurrence records collected between 26.04.2011 and 21.11.2023. They document the presence of 225,565 individual mosquitoes from 2,784 distinct locations. Of the total occurrences, 2,963 originate from citizen-science contributions, while the remainder stem from active monitoring. Passive monitoring was conducted through the citizen-science project “Mückenatlas”, in which volunteers collected and submitted mosquito specimens from across Germany. All citizen-collected specimens were subsequently identified by professional entomologists. Active monitoring was carried out by experts using a wide range of standardised entomological methods, including trapping, aspirating and netting adults, as well as sampling larvae and pupae by dipping and sieving. The dataset, or subsets thereof, has already been used in scientific publications.

The dataset submitted to GBIF is structured as an occurrence dataset following the Simple Darwin Core standard (version issued: 13.09.2023 Darwin Core Maintenance Group (2023)). The dataset and its associated metadata are available through the Pensoft IPT portal using the Integrated Publishing Toolkit (IPT), version 3.1.4 (Pothmann et al. 2026).

**Data set 1.** 

Column label	Column description
occurrenceID	An identifier for the dwc:Occurrence (as opposed to a particular digital record of the dwc:Occurrence). In the absence of a persistent global unique identifier, construct one from a combination of identifiers in the record that will most closely make the dwc:occurrenceID globally unique.
type	The nature or genre of the resource.
language	A language of the resource.
licence	A legal document giving official permission to do something with the resource.
rightsHolder	A person or organisation owning or managing rights over the resource.
accessRights	Information about who can access the resource or an indication of its security status.
references	A related resource that is referenced, cited or otherwise pointed to by the described resource.
datasetName	The name identifying the dataset from which the record was derived.
ownerInstitutionCode	The name (or acronym) in use by the institution having ownership of the object(s) or information referred to in the record.
basisOfRecord	The specific nature of the data record.
catalogNumber	An identifier (preferably unique) for the record within the data set or collection.
recordedBy	A list (concatenated and separated) of names of people, groups or organisations responsible for recording the original dwc:Occurrence. The primary collector or observer, especially one who applies a personal identifier (dwc:recordNumber), should be listed first.
organismQuantity	A number or enumeration value for the quantity of dwc:Organisms.
organismQuantityType	The type of quantification system used for the quantity of dwc:Organisms.
sex	The sex of the biological individual(s) represented in the dwc:Occurrence.
lifeStage	The age class or life stage of the dwc:Organism(s) at the time the dwc:Occurrence was recorded.
establishmentMeans	Statement about whether a dwc:Organism has been introduced to a given place and time through the direct or indirect activity of modern humans.
degreeOfEstablishment	The degree to which a dwc:Organism survives, reproduces and expands its range at the given place and time.
occurrenceStatus	A statement about the presence or absence of a dwc:Taxon at a dcterms:Location.
eventDate	The date-time or interval during which a dwc:Event occurred. For occurrences, this is the date-time when the dwc:Event was recorded. Not suitable for a time in a geological context.
verbatimEventDate	The verbatim original representation of the date and time information for a dwc:Event.
habitat	A category or description of the habitat in which the dwc:Event occurred.
higherGeographyID	An identifier for the geographic region within which the dcterms:Location occurred.
higherGeography	A list (concatenated and separated) of geographic names less specific than the information captured in the dwc:locality term.
continent	The name of the continent in which the dcterms:Location occurs.
country	The name of the country or major administrative unit in which the dcterms:Location occurs.
countryCode	The standard code for the country in which the dcterms:Location occurs.
stateProvince	The name of the next smaller administrative region than country (state, province, canton, department, region etc.) in which the dcterms:Location occurs.
locationAccordingTo	Information about the source of this dcterms:Location information. Could be a publication (gazetteer), institution or team of individuals.
decimalLatitude	The geographic latitude (in decimal degrees, using the spatial reference system given in dwc:geodeticDatum) of the geographic centre of a dcterms:Location. Positive values are north of the Equator, negative values are south of it. Legal values lie between -90 and 90, inclusive.
decimalLongitude	The geographic longitude (in decimal degrees, using the spatial reference system given in dwc:geodeticDatum) of the geographic centre of a dcterms:Location. Positive values are east of the Greenwich Meridian, negative values are west of it. Legal values lie between -180 and 180, inclusive.
geodeticDatum	The ellipsoid, geodetic datum or spatial reference system (SRS) upon which the geographic coordinates given in dwc:decimalLatitude and dwc:decimalLongitude are based.
verbatimLatitude	The verbatim original latitude of the dcterms:Location. The coordinate ellipsoid, geodeticDatum or full Spatial Reference System (SRS) for these coordinates should be stored in dwc:verbatimSRS and the coordinate system should be stored in dwc:verbatimCoordinateSystem.
verbatimLongitude	The verbatim original longitude of the dcterms:Location. The coordinate ellipsoid, geodeticDatum or full Spatial Reference System (SRS) for these coordinates should be stored in dwc:verbatimSRS and the coordinate system should be stored in dwc:verbatimCoordinateSystem.
georeferencedDate	The date on which the dcterms:Location was georeferenced.
verbatimIdentification	A string representing the taxonomic identification as it appeared in the original record.
identifiedBy	A list (concatenated and separated) of names of people, groups or organisations who assigned the dwc:Taxon to the subject.
scientificName	The full scientific name, with authorship and date information if known. When forming part of a dwc:Identification, this should be the name in lowest level taxonomic rank that can be determined. This term should not contain identification qualifications, which should instead be supplied in the dwc:identificationQualifier term.
taxonID	An identifier for the set of dwc:Taxon information. May be a global unique identifier or an identifier specific to the dataset.
kingdom	The full scientific name of the kingdom in which the dwc:Taxon is classified.
phylum	The full scientific name of the phylum or division in which the dwc:Taxon is classified.
class	The full scientific name of the class in which the dwc:Taxon is classified.
order	The full scientific name of the order in which the dwc:Taxon is classified.
family	The full scientific name of the family in which the dwc:Taxon is classified.
genus	The full scientific name of the genus in which the dwc:Taxon is classified.
subgenus	The full scientific name of the subgenus in which the dwc:Taxon is classified.
taxonRank	The taxonomic rank of the most specific name in the dwc:scientificName.

## Additional information

### Description of the dataset

**Figure 1.**
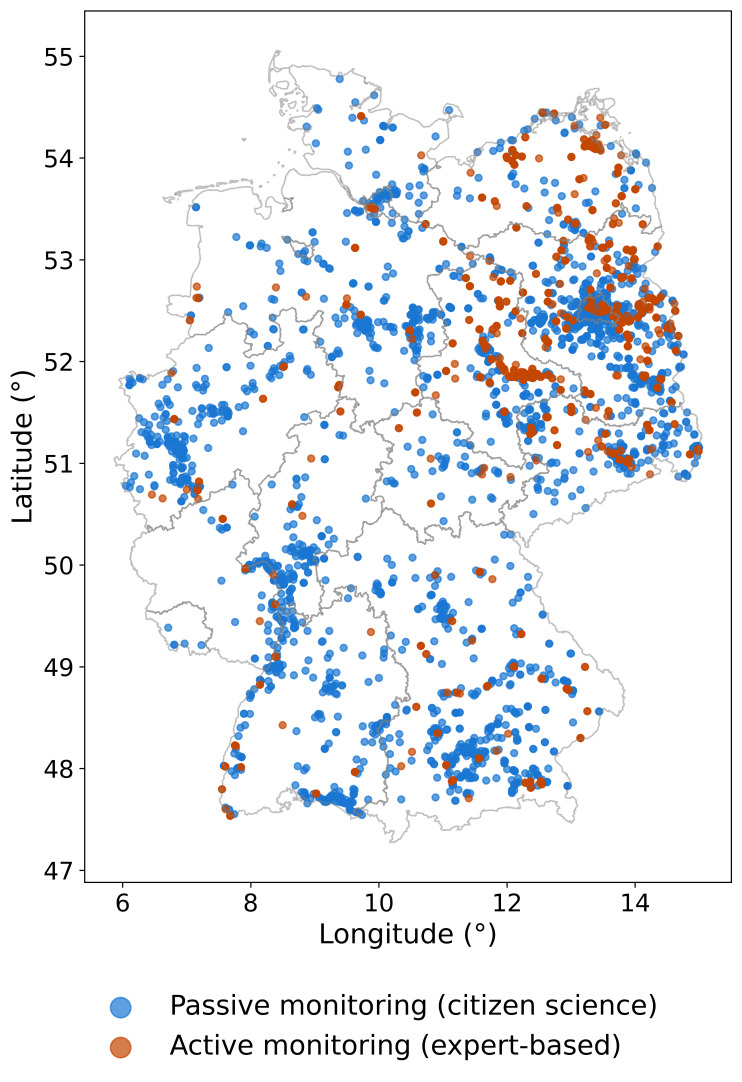
Presence occurrence record locations of *Ae. vexans* in Germany as used in this study.

Fig. 1 illustrates the spatial distribution of *Ae. vexans* occurrence records across Germany, as compiled here, differentiated by monitoring approach. The map demonstrates broad nationwide coverage. Active monitoring is predominantly concentrated in north-eastern Germany, reflecting the locations of the institutes conducting the surveys. Passive monitoring, on the other hand, mainly consists of submissions and observations from more densely populated areas. This spatial pattern indicates a potential sampling bias, as occurrence records are influenced by the geographic focus of active monitoring activities and by higher reporting probabilities in densely populated regions (Pernat et al. 2021).

**Figure 2.**
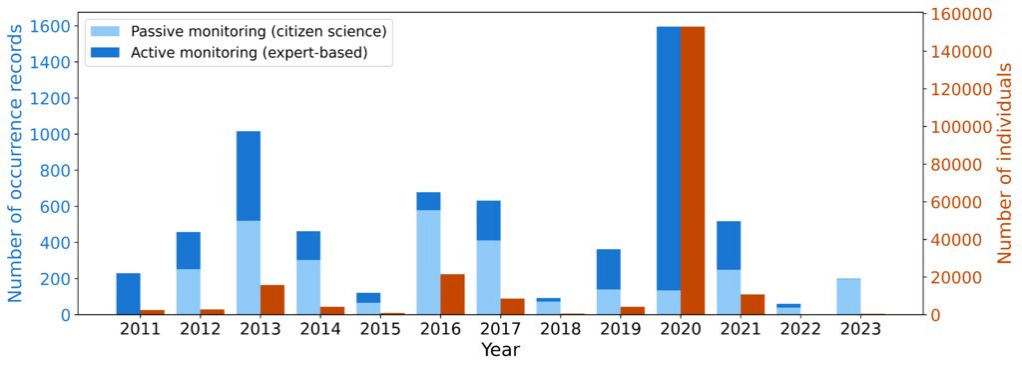
Number of *Ae. vexans* occurrence records and individuals per year.

Fig. 2 shows the annual number of occurrence records and individuals of *Ae. vexans*, differentiated by passive (citizen science) and active (expert-based) monitoring. The data reveal pronounced interannual variability, with notable peaks in 2013 and 2020, probably reflecting both natural fluctuations in mosquito populations and differences in sampling effort. Passive monitoring accounts for the majority of occurrence records, whereas active monitoring provides more consistent temporal coverage, but contributes fewer total records. Passive monitoring activities are driven by media coverage of mosquitoes (Pernat et al. 2022). The number of recorded individuals exhibits extreme peaks, particularly in 2020, suggesting mass occurrence events. However, sampling intensity was also highest in 2020, indicating that the observed peak is at least partly driven by increased monitoring efforts. Overall, the temporal patterns in the dataset point to potential sampling bias, as variation in sampling intensity influence the number of recorded occurrences (Pernat et al. 2021).
